# Temperature Analyses in Fused Filament Fabrication: From Filament Entering the Hot-End to the Printed Parts

**DOI:** 10.1089/3dp.2020.0339

**Published:** 2022-04-11

**Authors:** Jie Zhang, Egle Vasiliauskaite, Alec De Kuyper, Cédric De Schryver, Frederik Vogeler, Frederik Desplentere, Eleonora Ferraris

**Affiliations:** ^1^ Department of Mechanical Engineering, KU Leuven, Leuven, Belgium.; ^2^ Campus De Nayer, Thomas More University of Applied Sciences, Sint-Katelijne-Waver, Belgium.; ^3^ Department of Materials Engineering, KU Leuven, Leuven, Belgium.

**Keywords:** efficiency, fused filament fabrication, robo-FDM, scalability, single-walled structure, volume flow rate limit

## Abstract

This article analyzes temperature fields and their variations in fused filament fabrication (FFF) from the filament entering the hot-end to the printed parts, aiming at a deeper understanding of the thermal process of this additive manufacturing technology. A standard E3D print head assembly was mounted on a robot arm for printing. A stable filament feeding region was determined with an upper limit in the volume flow rate at different nozzle temperatures. Within the limit, the steady-state temperature fields inside the hot-end were studied by a computational fluid dynamics model. Simulations indicated that the temperature became less homogeneous at higher flow rates, leading to a lower extrudate temperature at the nozzle outlet. These outlet temperatures were analyzed, validated, and used as input to simulate temperature variations in printed parts with a self-developed open-access numerical model. An interlayer time similarity rule was found in printing single-walled geometries, which specifies temperature similarities at the same interlayer time. The findings provide new insights into FFF processes, pointing out opportunities for improved production efficiency and scalability to large-scale manufacturing.

## Introduction

Extrusion-based additive manufacturing (AM) includes a variety of manufacturing technologies.^1^ A big sub-branch of them is driven by thermal energy, such as fused filament fabrication (FFF), pellet-based hot-melt extrusion,^2^ big area additive manufacturing,^3^ molten soda-lime glass extrusion,^4,5^ and molten sugar AM.^6^ For these technologies, an in-depth knowledge of temperature development during printing is the key to fully understand the manufacturing process and to identify the limitations and capabilities.

**FIG. 1.**
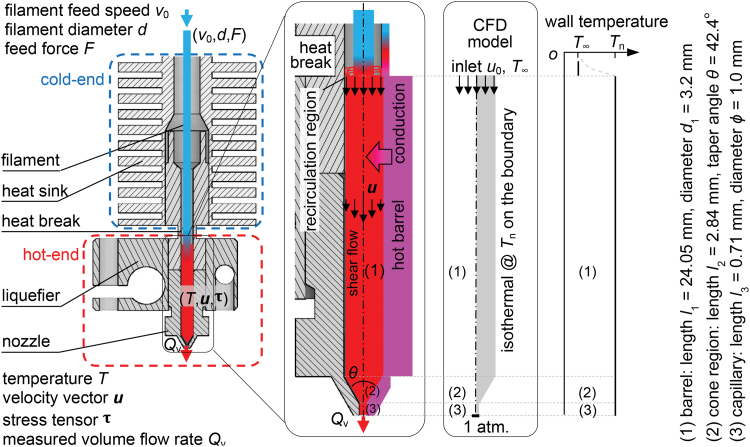
A print head assembly in FFF, and the polymer melt flow, pressure and heat transfer inside the hot-end. The hot-end was modeled in the CFD model for three geometrical parts: (1) barrel, (2) cone, and (3) capillary end. The barrel includes part of the liquefier and part of the nozzle. FFF, fused filament fabrication. CFD, computational fluid dynamics. Color images are available online.

In FFF, the temperature development starts from a thermoplastic filament feedstock being typically driven by a pinch roller, and fed into a hot-end consisting of a liquefier and a nozzle (Fig. 1). In the liquefier, the filament is heated up by a hot barrel, mainly by thermal conduction, toward a nominal nozzle temperature $T_{n}$. In the meantime, the polymer melt spreads and fills in the barrel, and flows under pressure. At the steady state, the small gap between the barrel and filament at the hot-end entry is sealed by a recirculation region of polymer, which has an intermediate temperature between the material glass transition temperature $T_{g}$ and the nozzle temperature $T_{n}$ (Fig. 1).^7,8^ Hence, the feeding pressure is transferred from the filament in the cold-end to the polymer melt inside the liquefier. The recirculation region typically locates at the heat break,^8^ where there exists a sharp temperature gradient from $T_{n}$ in the hot-end to the room temperature $T_{\infty }$ in the cold-end (Fig. 1). This recirculation vortex may move downward at a higher filament feeding speed, leading to a reduction in the contact area for conductive heat transfer. Such a change can exacerbate the mismatch between the designed and actual energy transfer rate from the hot-end barrel to the polymer melt flow,^8^ and may explain the well-observed transitions from the stable to unstable filament feeding.^8–10^

Then, the polymer melt in the barrel is pushed through the nozzle, where it is subjected to an extensional flow because of the reduced cross-sectional area of the cone (conical/contraction) region and capillary end of the nozzle.^11^ Shortly afterward, it reaches the exit and leaves the nozzle tip. The distributions and variations of the polymer melt velocity, pressure, and temperature, from the barrel entrance to the nozzle exit, are topics of intensive researches.^9,10,12–20^ On the one hand, these physical fields are typically affected by the liquefier temperature and feed rate for a given hot-end assembly and filament material^10,13–15^; on the other hand, the geometrical characteristics of the liquefier and the nozzle (e.g., length of the barrel, taper angle of the cone, diameter of the capillary end) also play a significant role.^9,10,19,20^

After leaving the hot-end, the deposition happens: the polymer melt makes a 90° turn, and typically deforms from a circular cross section to an ellipse confined between the nozzle tip and the build (support/heat) plate. During the deformation, the bottom side of the strand (track/road/bead) experiences profound stretches.^21^ Such stretches, along with the shear flow inside the hot-end, may significantly disentangle the polymer chains and accelerate the crystallization kinetics for semicrystalline polymers.^22^ Accompanying the deposition, heat transfer takes place in the strand due to a combination of thermal conduction (with previously deposited neighbor strands and/or the build plate if applicable), convection (with the local air), and radiation (with the far environment).^3,23^ The printed part solidifies as its temperature decreases toward the room temperature $T_{\infty }$. During the cooling phase, reheating may happen due to thermal contacts with newly deposited neighbors on the same layer (intralayer reheating), and/or from the layer above (interlayer reheating),^24^ and/or due to the reheatings of these neighbors (secondary reheatings). Melt-crystallization may also happen (for semicrystalline materials) due to the aforementioned shear flow-induced disentanglement and/or material composition modification,^25^ and it may ultimately affect the overall cooling rate and the degree of crystallinity in printed parts.^26^

As introduced, temperature analyses in FFF are usually studied in the above two disjointed stages inside and outside the hot-end. To the best of the authors' knowledge, no studies provide a complete description of the temperature development from the filament to the final parts yet.

This article offers such a global view. The aim is to reach a deeper understanding of the whole FFF process from the perspective of temperature development, and to investigate possible connections between liquefier kinetics and process parameters in the deposition. A print head assembly used on desktop FFF machines was mounted on a robot arm, and experiments in different processes were performed. The polymer melt flow, pressure, and heat transfer inside the hot-end were studied with a computational fluid dynamics (CFD) model. The extrudate temperatures at the nozzle outlet were analyzed, validated, and then used to simulate part cooling during printing by means of a self-developed finite difference model. The temperature variations in the printed parts were studied subsequently, along with the in-process monitoring by thermography. Accordingly, limitations across nozzle temperature, flow rate, and process parameters were identified. Suggestions for scale-up production and FFF process optimizations were also put forward.

## Materials and Methods

### Equipment and filament

**FIG. 2.**
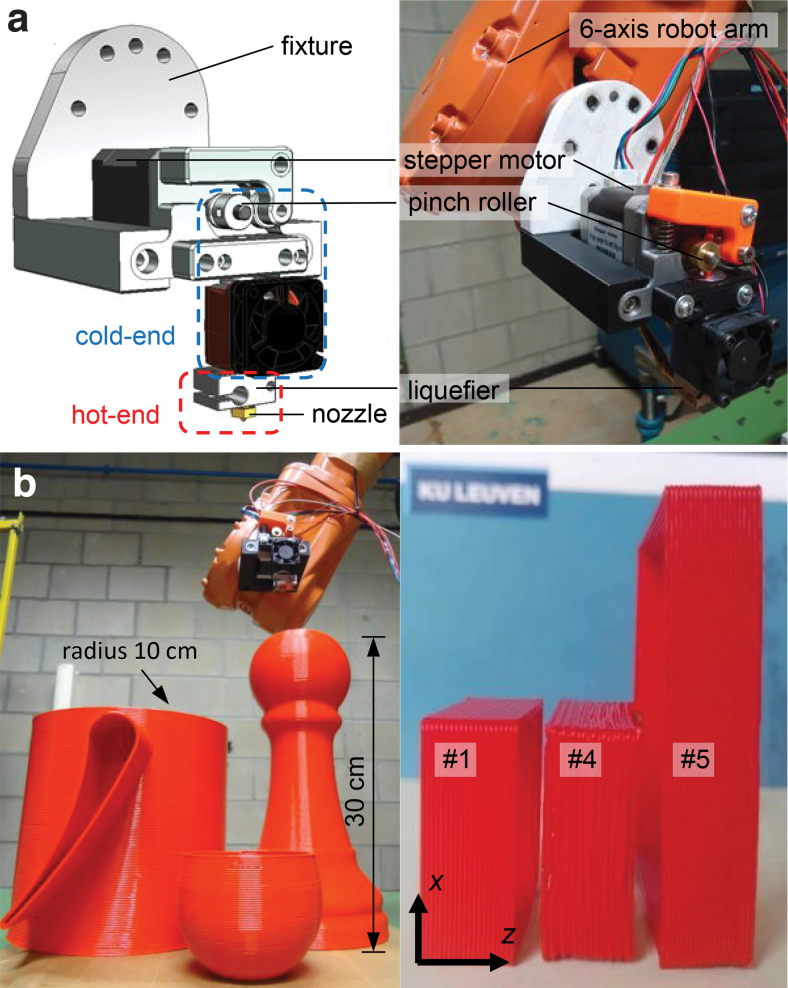
**(a)** The robot arm assisted FFF setup. *Left*: the CAD model of the print head assembly and the fixture; *right*: a picture of the actual setup. **(b)** Pictures of parts printed with the robot arm assisted FFF setup. *Left*: examples of printing big parts and printing on curved surfaces; *right*: single-walled samples printed under the investigation. Color images are available online.

A standard E3D v6 print head with an E3D brass nozzle (diameter *ϕ* = 1 mm) was used (Fig. 2a). A Nema 17 stepper motor with a holding torque of 0.49 N·m was used to feed the filament. The liquefier was powered by a ceramic heating element with a maximum power of 30 W, and its temperature was measured by a K-type thermocouple. The temperature and extrusion rate of the liquefier were controlled by an embedded PC (CX5140; Beckhoff Automation GmbH & Co. KG, Germany) with appropriate input/output cards (stepper-EL7041-0052, heating element-EL2022, thermocouple-EL3312) in a custom program written in the TwinCAT 3 software. The function block TF4110 TC3 Temperature Controller was used to periodically switch on and off the heating element with the digital pulse-width modulation signal. The modulation cycle time was 1 s. During the extrusion, the difference between the nominal nozzle temperature $T_{n}$ and the measurement by the thermocouple was below 0.1℃.

The print heat assembly was mounted on a six-axis robot arm with a maximum reach of 1.55 m (ABB IRB 2400; ABB Ltd., Switzerland, Fig. 2a) and controlled for printing. The printing toolpaths were programmed in ABB RobotStudio^®^ 2019 (ABB Ltd.) program.

The material used was a red ColorFabb poly(lactic acid) (PLA) Economy filament with a diameter *d* = 2.85 mm. When tested by differential scanning calorimetry (TA Instruments Q200, heating rate 10℃/min), its degree of crystallinity was found to be below 1% (in the first heating cycle), indicating that the filament was in an amorphous state before being used in FFF.

### Geometry and process settings

**FIG. 3.**
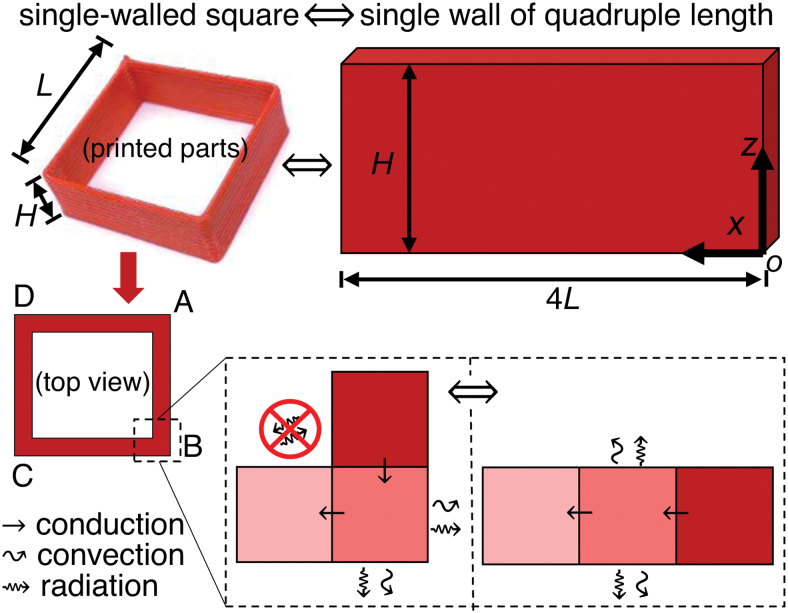
The geometry of the single-walled square in this study. From the perspective of heat transfer mechanisms, the geometry is equivalent to a single wall of a quadruple length in the simulations when the radiant heat transfer between different locations is ignored (drawing not to scale). Color images are available online.

The geometry of interest is a single-walled square of length *L* = 30 mm, height *H* = 12 mm, and wall thickness 1 mm (Fig. 3). This geometry provides a straightforward visual means to identify a suitable process window for quality printing. It is also of practical applications in contouring of large single-walled structures. From the perspective of thermal boundaries in heat transfer, it represents a worse scenario for bond formation due to fast cooling. Thus, the temperature obtained with this geometry also sheds lights on the weak bonding in FFF.

Experiments were conducted at different nozzle traveling speeds *v*, this being an important parameter in high-throughput printing, which affects not only the printed part quality but also the deliverable maximum volume flow rate. Four flow conditions were investigated in printing samples 1 to 4 at different *v* (Table 1). Each *v* determined a designed volume flow rate $Q_{v}^{\prime }$ as such

**Table 1.** Samples, Dimensions, and Process Parameters in Fused Filament Fabrication

Sample No.	Edge length	Nozzle traveling speed	Designed volume flow rate $Q_{v}^{\prime }$	Interlayer time Δτ	Filament feed speed
	L [mm]	v [mm/s]	[cm^3^/h]	[s]	v*_0_* [mm/min]
1	30	5.0	14.2	24	37
2	30	7.5	21.4	16	56
3	30	10.0	28.3	12	74
4	30	13.5	38.3	8.89	100
5	60	10.0	28.3	24	74

(1) $$Q_{v}^{\prime }=Av,$$

where *A* is the real strand cross-section area. *A* can be expressed by

(2) A=ξϕ¯h,

where ¯h is the layer height (layer thickness), ϕ the nozzle diameter, and ξ the strand shape factor. The product ξϕ is also understood as the strand width, which means ξ is an independent variable in FFF (thus making *A* and $Q_{v}^{\prime }$ dependent variables). ξ was set 1.3 in this study, ¯h was set to be 0.6ϕ, that is, ¯h=0.6 mm. In addition, the nominal nozzle temperature $T_{n}$ was set to 200℃. The build plate was a plastic board covered with paper tape at the room temperature ($T_{\infty }=$25℃).

For comparison, sample 5 of a doubled edge length *L*=60 mm was printed at v=10 mm/s, so that it shared the same interlayer time Δτ as sample 1. Δτ here is defined as the time required to print one entire layer, and therefore also the time gap between two deposited elements of material on the top of each other. For this single-walled square, Δτ=4L∕v. Δτ was 24 s for both samples 1 and 5.

### Flow rate investigations

The volume flow rate is a vital efficiency index in FFF. The faster the material flows through the hot-end, the faster the deposition, and the shorter it takes to finish the printing. The limits in the flow rate for the given print head assembly were investigated at different filament feed speeds *v*_0_ and nozzle temperatures $T_{n}$. Three nominal $T_{n}$ were used: 190℃, 200℃, and 210℃. Theoretically, *v*_0_ is determined by the ratio between the designed volume flow rate and the filament cross-section area ($\pi d^{2}∕4$):

(3) $$v_{0}=\frac{4Q_{v}^{\prime }}{\pi d^{2}}.$$

In practice, irrespective of $T_{n}$, *v*_0_ is realized by the pinch roller. However, the print head may fail to accurately deliver the required $Q_{v}^{\prime }$. Thus, the actual volume flow rate was measured: the hot-end was set to continuously extrude for 120 s, the extruded mass was weighed, and the mass flow rate $Q_{m}$ calculated assuming a constant flow rate. Then, the measured volume flow rate $Q_{v}$ was calculated by the following:

(4) $$Q_{v}=\frac{Q_{m}}{\rho_{0}},$$

where $\rho_{0}=$1.25 g/cm^3^ is the density of PLA at 25℃. Under each test condition, three measurements on $Q_{v}$ were taken, and the arithmetic means were reported with the standard deviations, in contrast to their designed values $Q_{v}^{\prime }$.

### Simulations inside the hot-end

The polymer melt flow, pressure, and heat transfer inside the hot-end fundamentally determine the fused deposition rate and the upper limit in extrusion. These physical fields were solved by a 2D axisymmetric CFD model taking into account conduction, convection, shear heating, and possible slip at wall effects due to high local shear stresses. The hot-end in the model consists of three geometrical parts: (1) a barrel of length *l*_1_ and diameter *d*_1_, (2) a cone region of length *l*_2_ and taper angle θ, and (3) a capillary end of length *l*_3_ and diameter ϕ (Fig. 1). The transport equations for mass, momentum, and energy are as follows:

(5) $$\frac{\partial \rho }{\partial t}+\nabla \cdot \left(\rho u\right)=0,$$

(6) $$\frac{\partial \left(\rho u\right)}{\partial t}+\nabla \cdot \left(\rho uu\right)=-\nabla p+\nabla \cdot \tau ,$$

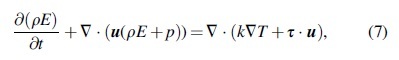


where ρ denotes the mass density, ***u*** the velocity vector, *p* the pressure, *k* the thermal conductivity, τ the stress tensor, *T* the temperature, and *E* the sum of enthalpy and kinetic energy density,$E=\int_{T_{\infty }}^{T}c_{p}dT+\frac{u^{2}}{2}$, in which *c_p_* is the specific heat capacity at constant pressure, and $T_{\infty }$ the room temperature. The stress tensor τ=2ηD, with $D=\frac{1}{2}\left(\nabla u+{\left(\nabla u\right)}^{T}\right)$, denoting the rate of deformation tensor and η the non-Newtonian shear viscosity. The viscoelastic effects were neglected together with the initial degree of crystallinity. Besides, the cold crystallization of PLA was also neglected because its crystallization half time is considerably longer than the typical residence time of the polymer inside the hot-end^25^; even if it happened, the energy released would eventually be absorbed during the melting,^11^ giving a net-zero contribution to the temperature field in a state above the melting temperature.

The Ansys Fluent (version R 19.0) software was used to solve the coupled CFD model at the steady state. Before the simulation, material properties in Equations (5)–(7) must be available. For PLA, *c_p_* and ρ took the temperature-dependent form from literature.^27,28^ A constant thermal conductivity was taken, k=0.2 W/m/℃.^28,29^ The shear viscosity η was measured in an Anton Paar MCR 102 parallel plate rheometer. Relevant details are given in Supplementary Appendix SA1.

At the steady state, the polymer melt was assumed to fill the whole barrel and driven by the filament from the cold-end. At the barrel entrance, an adjusted uniform inlet speed $u_{0}=v_{0}d^{2}∕d_{1}^{2}$ was assumed due to the apparent difference between the barrel diameters *d*_1_ and filament diameter *d*. The inlet speed *u*_0_ is connected to the dimensionless Péclet number^13^ Pe as such

(8) $$Pe=\frac{\rho c_{p}u_{0}d_{1}^{2}}{4k\left(l_{1}+l_{2}\right)}=\frac{Q_{v}^{\prime }}{\pi \left(l_{1}+l_{2}\right)\alpha },$$

where $\alpha =\frac{k}{\rho c_{p}}$ is the thermal diffusivity, $Q_{v}^{\prime }=\frac{u_{0}\pi d_{1}^{2}}{4}$, and $\left(l_{1}+l_{2}\right)$ denotes the total length in the barrel and cone regions (Fig. 1), characterizing the effective area for thermal conduction. Pe describes the competition in heat transfer between the convection and conduction inside the hot-end, and provides a versatile measure for $Q_{v}^{\prime }$ (Table 2).

**Table 2.** Simulation Details and Results Inside the Hot-End

Sample No.	Designed volume flow rate $Q_{v}^{\prime }$	Inlet speed u_0_		Feed force F	ΔT ^a^	$\max\left|\partial T∕\partial r\right|$	Deposition temperature $\bar{T}$
	[cm^3^/h]	[mm/min]	Péclet number Pe	[N]	[℃]	[℃/mm]	[℃]
1	14.2	29.3	0.58	3.81	0.4	1.25	199.8
2	21.4	45.2	0.88	5.53	2.7	7.65	198.6
3	28.3	58.7	1.16	7.23	6.8	19.05	196.5
4	38.3	79.3	1.57	9.64	15.2	42.20	192.1

^a^ ΔT denotes the difference between the temperature at the center and at the wall in the extrudate at the nozzle outlet.

The filament was assumed to enter the barrel at room temperature 25℃. Simulations at higher filament inlet temperature were also conducted, showing negligible impact on the output. The walls in the hot-end (barrel, cone, and capillary) were set isothermal at $T_{n}$ (200℃, Fig. 1). Under the pressure exerted by the filament, the polymer melt slipped at the walls (nonzero velocity condition). The slip velocity $u_{s}$ was included by a user-defined function according to the slip law^30^

(9) $$u_{s}=u^{*}{\left(\frac{\tau_{w}}{\tau_{r}}\right)}^{n_{s}},$$

where $\tau_{w}$ is the shear stress at the wall, $\tau_{r}$ a reference stress, $n_{s}$ the power index, and $u^{*}$ the slip velocity at the reference stress. The material-specific slip parameters $\tau_{r}=$100 kPa, $n_{s}=$2, and $u^{*}=$5 mm/s were used. They have not been measured for the PLA filament, but can be regarded as reasonable values from the range of polymer melts with known slip parameters.^31^ The uncertainties in these parameters can lead to considerable variations in the velocity and pressure fields, but the temperature profiles will remain largely unaffected. At the outlet, the pressure was set at the atmospheric value.

The extrudate temperature at the nozzle outlet/deposition temperature $\bar{T}$ was defined as the mass-weighted mean temperature as such

(10) $$\bar{T}=\frac{\int T\rho u\cdot dA}{\int \rho u\cdot dA},$$

where *T* is the local temperature at the nozzle outlet, ρ the mass density, ***u*** the exit velocity vector, and dA the cross-sectional area element. This mean temperature was used as the initial condition for temperature simulations in the printed parts, as the Biot number $Bi=\frac{h¯h}{\lambda }=$0.026 is <<1 (*h* takes 8.5 W/m^2^/K as the coefficient of natural convection^3^), indicating a uniform temperature distribution across the strand cross section.

### Temperature simulations in printed parts

The same set of transport Equations (5)–(7) were used to describe the physics in the printed parts. When assuming that the velocity u=0 with reference to the build plate, the partial differential equation for energy conservation decoupled from the mass and momentum conservations, and Equation (7) took the form of

(11) $$\frac{\partial T\left(x,z,t\right)}{\partial t}=\alpha \nabla^{2}T,\left(x,z\right)\in \Omega \left(t\right).$$

The material properties ρ, *c_p_*, and *k* defining the thermal diffusivity α ($=k∕\left(\rho c_{p}\right)$) took the same values as in the previous section. The geometry $\Omega \left(t\right)$, when finished, was the single wall of quadruple length as demonstrated in Fig. 3. Equation (11) did not include the volumetric heat generation by possible melt crystallization of PLA during cooling, which is reasonable due to the slow crystallization kinetics^25^ and necessary because of the complexity in the nonlinear heat generation. In addition, Equation (11) also ignored the material anisotropy in thermal conduction.

The temperature simulation for the printed parts was solved as an initial-boundary value problem for thermal conduction on the changing domain $\Omega \left(t\right)$. The strand cross section was assumed to be a rectangle of width ξϕ and height ¯h. The boundary condition (BC) took the combined form of thermal convection and radiation, where the radiant heat flux was assumed to be hemispherical (ignoring the radiant heat transfer between different locations, Fig. 3). The total heat flux along the outward normal of a free surface is as follows:

(12) $$q=q_{conv}+q_{rad}=h\left(T-T_{\infty }\right)+\varepsilon \sigma \left(T^{4}-T_{\infty }^{4}\right),$$

where *h* is the convection coefficient, σ= 5.670×10^−8^ W/m^2^/K^4^ the Stefan–Boltzmann constant, and ε the material emissivity. ε=0.78 was taken from the wavelength-specific emissivity $\varepsilon_{\lambda }$ in the thermography,^24^ and h=8.5 W/m^2^/K for natural convection. Particularly, the temperatures in Equation (12) took the unit in Kelvin (K). On the bottom side of the first layer, the Dirichlet BC was used, where the plate temperature was assumed equal to the room temperature $T_{\infty }$.

A boundary-adjusting finite difference method was developed for the simulation. The numerical model is available from the Advanced Manufacturing Lab,^32^ by means of which all simulations for printed parts in this work can be reproduced.

### Temperature monitoring

An infrared (IR) thermal camera (Optris PI640) was used to monitor the temperature fields and their variations in the printed parts once the polymer melt left the nozzle. In particular, the temperature upon deposition was captured and compared with the CFD simulations. The camera worked in the long-wavelength spectrum range of 7.5–13 μm, and recorded temperature fields at a spatial resolution of 31.25 μm/pixel and a frequency of 32 Hz. The measurement error was ±2℃ or ±2%, whichever was greater.

The field of view was in the center of edge BC (Fig. 3). The temperature assigned at a given location took the arithmetic mean of the temperatures measured on the surrounding 6 × 3 pixels. With this IR camera, the emissivity of the printed PLA $\varepsilon_{\lambda }$ was forced to 1, to avoid an overestimation in the deposition temperature. The same emissivity choice was also used in literature.^11^ Although the absolute accuracy of IR thermography in FFF remains doubtful (due to changing geometry, thermal reflections, calibration, etc.), the recorded temperatures are informative and suitable for comparison purposes.

This study is exempt from the institutional review board for approval.

## Results and Discussions

### The limit in the volume flow rate

**FIG. 4.**
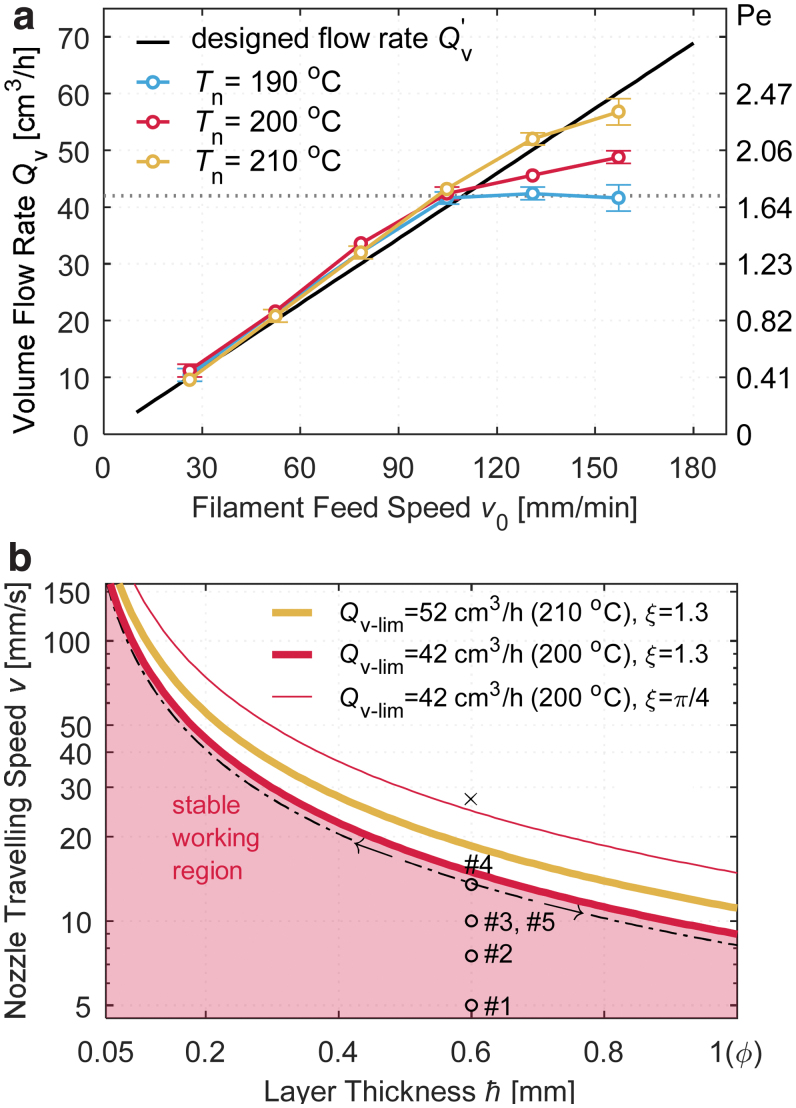
**(a)** Relationship between the filament feed speed *v*_0_ and the measured volume flow rate $Q_{v}$ at different nozzle temperatures $T_{n}$. **(b)** The stable working region of the hot-end in terms of layer thickness ¯h and nozzle traveling speed *v*, given the volume flow rate limit $Q_{v-lim}$ at different nozzle temperatures and the strand cross-section shape factor ξ. Color images are available online.

Fig. 4a shows the measured volume flow rates $Q_{v}$ at each filament feed speed *v*_0_ and nozzle temperature $T_{n}$, in contrast to their designed values $Q_{v}^{\prime }$. Irrespective of $T_{n}$, $Q_{v}$ can follow $Q_{v}^{\prime }$ at a relatively low *v*_0_ (e.g., when $v_{0}\leq$100 mm/min), although with some slight deviations. When increasing *v*_0_, a limit volume flow rate $Q_{v-lim}$ can be observed, beyond which, $Q_{v}$ no longer follows the designs. This limit manifests the transition from stable to unstable feeding in FFF,^8,9^ and depends on the nozzle temperature $T_{n}$, material properties, and the filament feeding machinery. Specifically, a $Q_{v-lim}=$42 cm^3^/h was observed when $T_{n}$ was 200℃ (and 190℃); it extended to 52 cm^3^/h at 210℃ in this study. Such temperature dependence agrees with the observations on the liquefier dynamics that a higher nozzle temperature benefits the pressure transfer and allows a higher material deposition rate.^33,34^

This transition from the stable to unstable polymer melt flow can also be described by the Péclet number [Eq .(8)]. At $T_{n}=$200℃, the limiting Pe for $Q_{v-lim}=$42 cm^3^/h was 1.72; at $T_{n}=$210℃, the Pe limit was 2.13. These limits are on the same magnitude as those reported by Luo *et al.*^13^ (0.89) and Serdeczny *et al.*^8^ (0.5–1), despite all the differences in the machines and materials. Particularly, when printing samples 1 and 2, Pe<1 (Table 2), indicating that the thermal conduction is in a more predominant position, and thus, the temperature gradients at the nozzle outlet should be small; when printing samples 3 and 4, Pe>1, higher temperature gradients are anticipated. These anticipations are in line with the exact conclusions by Luo *et al.*^13^

Once a $Q_{v-lim}$ is determined, a few process parameters are bounded accordingly. To ensure that the hot-end works in a reliable state, the nozzle traveling speed *v* must obey

(13) $$vA\leq Q_{v-lim}.$$

Substituting Equation (2) for the strand cross-section area *A*, the inequality (13) then suggests the following:

(14) $$¯hv\leq \frac{Q_{v-lim}}{\xi \phi },$$

which means that the nozzle traveling speed *v* and layer height ¯h are bounded above within a stable working region as shown in Fig. 4b, disregarding their limits set by the mechanical motion system or axis resolution. Particularly, it is observed that at a smaller layer height ¯h, the traveling speed *v* can be higher while keeping the same volume flow rate. This result suggests that choosing a smaller layer height in printing does not necessarily mean a lower production efficiency. Besides, with a smaller strand shape factor ξ (a smaller strand width) or a higher nozzle temperature, both ¯h and *v* can be higher. Fig. 4b also plots the v−¯h coordinates of the five samples in this work: they are all located in the stable region, and thus, the designed process conditions are reliable.

The limiting $Q_{v-lim}$ in Fig. 4a originates from the transition from the stable to unstable filament feeding in the hot-end. Thus, this limit is more restrictive than the limit by nozzle clogging^13,15^ or the slippage in the filament feeding. It is worthwhile to denote that the FFF process parameters outside the stable region could work as well, despite the process control being more complex or an underextrusion will take place without countermeasures. Nevertheless, process parameters inside the stable region will not guarantee a successful printing, as factors such as cooling rates can also lead to printing failures.

### Simulations inside the hot-end

At different volume flow rates $Q_{v}^{\prime }$ ( = $Q_{v}$ in the stable region), the velocity, stress, and temperature fields were solved from the coupled CFD model.

When the stress was integrated over the whole internal surface in the hot-end, the feed force exerted on the filament *F* was obtained, and is summarized in Table 2. Logically, *F* increased with the volume flow rate $Q_{v}^{\prime }$. These forces agree with the publications^11,13,16^ in the magnitude, but are generally lower because of the negative dependence of the pressure on the nozzle diameter: in this study, ϕ=1 mm is higher than that used in the mentioned publications, and thus, the pressure accumulation inside the nozzle is accordingly lower. At the maximum $Q_{v}^{\prime }$, the feed force was only about 10 N, which was in a safe region for the stepper motor, as it can theoretically exert a maximum force of 98 N at a gear radius of 5 mm. Despite the simulations being subjected to considerable variations as aforementioned, the printing is safe from nozzle clogging.

**FIG. 5.**
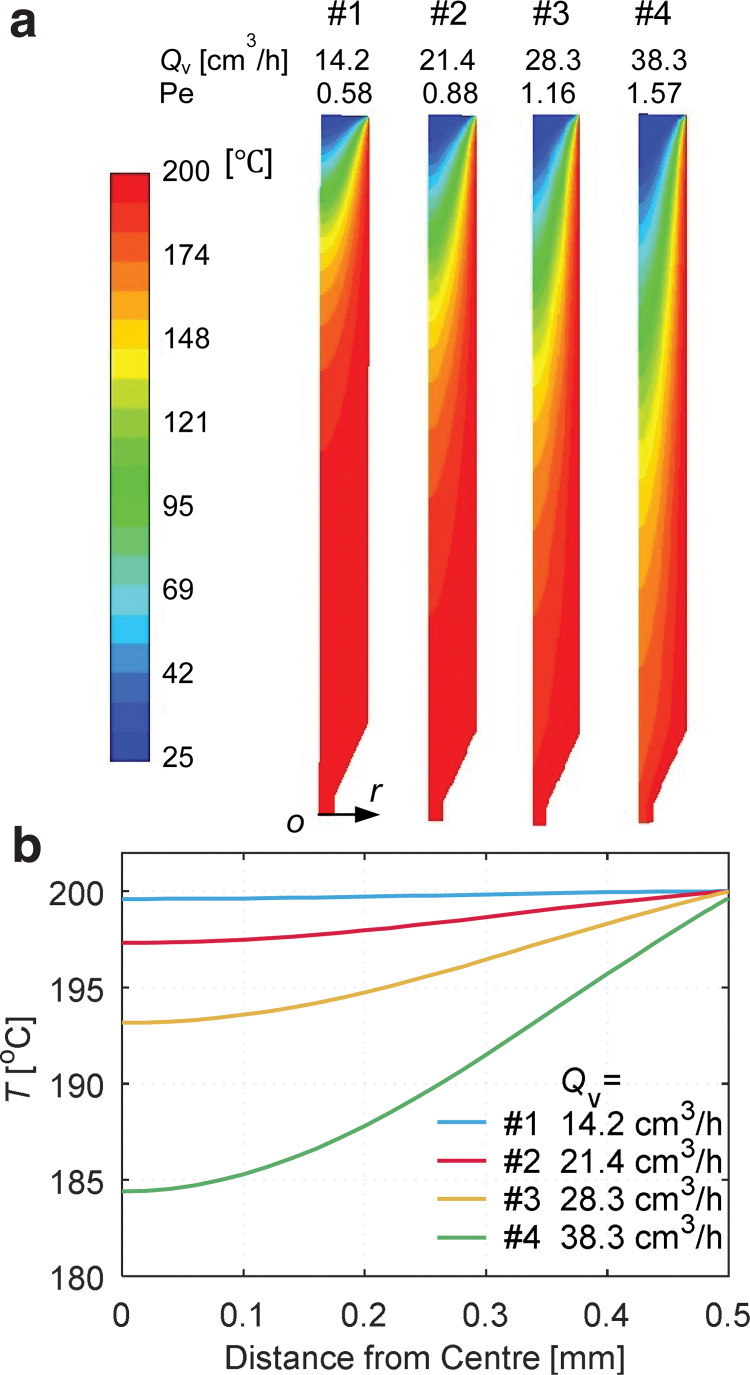
**(a)** Temperature fields in the hot-end at different volume flow rates $Q_{v}$. **(b)** Radial temperature distribution at the nozzle outlet. Color images are available online.

In the four flow conditions for each sample, the steady-state temperature fields inside the hot-end are shown in Fig. 5a. In all cases, the temperature fields are highly nonisothermal. At the entrance, the temperature was $T_{\infty }$, specified by the BC. The higher the $Q_{v}$ (or Pe), the further the polymer's cold core traveled inside the hot-end. The polymer contacting the isothermal wall had a similar temperature as the nominal nozzle temperature, but its temperature decreased toward the center. In the whole barrel, considerable temperature gradients existed in the radial direction; but inside the cone and the capillary end, the gradients were much milder.

The radial temperature distributions at the nozzle outlet are shown in Fig. 5b. Let ΔT denote the difference between the polymer temperature in the center and at the wall. ΔT was below 5℃ under flow conditions for samples 1 and 2 when Pe<1 (Table 2), which agrees with the analytical results in Luo *et al.*^13^ ΔT was 6.8℃ for the flow rate for sample 3 at a Pe of 1.16; ΔT reached 15.2℃ for sample 4 when Pe increased to 1.57, suggesting that at a higher flow rate, the polymer in the center did not receive enough thermal energy by conduction.

Another characteristic of the temperature field is the maximum temperature gradient intensity, $\max\left|\partial T∕\partial r\right|$. Their values for the four flow conditions are presented in Table 2 as well. When Pe was low (e.g., <1), such gradient intensity can be neglected, and the radial temperature distributions were quite homogeneous (Fig. 5b); however, at Pe=1.57 in printing sample 4, the maximum gradient reached 42.20℃/mm, and the profile exhibited considerable variations. These results confirm the anticipations in the previous section. If Pe or $Q_{v}$ further increased, both ΔT and $\max\left|\partial T∕\partial r\right|$ would increase accordingly. Eventually, clog will happen when the extrudate temperature at the nozzle outlet drops to a level below a threshold temperature defined by the melting temperature *T*_m_^13^ or the glass transition temperature $T_{g}$.^15^

The profiles in Fig. 5b were used in Equation (10) to calculate the extrudate temperature at the nozzle outlet $\bar{T}$. In four simulations, $\bar{T}$ was rather close to the nominal nozzle temperature $T_{n}$ (200℃) when the hot-end worked in the stable feed region (Table 2). $\bar{T}$ decreased slightly with the volume flow rate $Q_{v}^{\prime }$, from 199.8℃ at 14.2 cm^3^/h to 192.1℃ at 38.3 cm^3^/h. At low $Q_{v}^{\prime }$, the absolute differences between $\bar{T}$ and $T_{n}$ were negligible, but they extended to 7.9℃ at 38.3 cm^3^/h. Despite so, the deposition temperatures were sufficiently higher than the melting temperature of PLA (∼160℃).

**FIG. 6.**
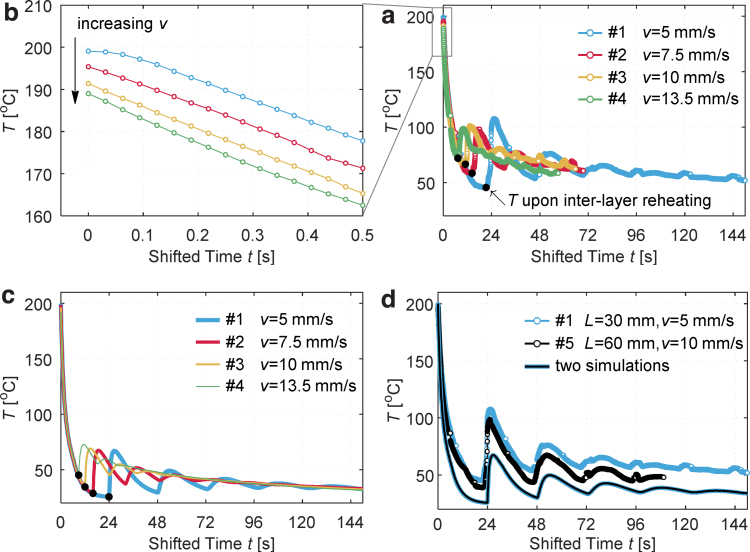
Temporal temperature profiles at different nozzle traveling speeds *v*. **(a, b)** Monitoring and **(c)** simulations. The monitoring and simulations confirm that at a higher *v* (a higher volume flow rate accordingly), the extrudate temperature at the nozzle outlet is lower. The *black dots* in **(a, c)** indicate the temperatures of material before the interlayer reheating. These temperature are higher at higher *v*. **(d)** Temporal temperature profiles in printing sample 1 (L=30 mm, v=5 mm/s) and 5 (L=60 mm, v=10 mm/s) when the interlayer time is the same. Color images are available online.

These average temperatures at different flow rates were verified by the IR camera when printing different samples: the same trend in the extrudate temperature reduction with increasing volume flow rate was observed. At 14.2 cm^3^/h (in printing sample 1), the temperature reduction can be ignored (Fig. 6a, b); at 38.3 cm^3^/h, the absolute difference was 11.0℃. (Considering the emissivity uncertainty in IR camera, the temperature reduction in printing sample 4 was 10.1℃ when compared with sample 1.) The observations partially verified the temperature results from CFD simulations. It is important to note that the extrudate temperature at the nozzle outlet $\bar{T}$ can significantly deviate from $T_{n}$ at higher $Q_{v}$. A deviation up to 30℃ was observed at a flow rate of 46.3 cm^3^/h (Pe=1.90). Deviations by 20–30℃ were also reported in literature.^8,13^ In those cases, melt fractures and sharkskin may accompany the temperature reduction, which ultimately affect the surface quality, part cooling, and bond strength.

To conclude hereby, both the feed force and extrudate temperature at the nozzle outlet suggest that nozzle clogging is far from being a problem in this study, which means the limit by nozzle clogging is less useful in guiding process parameter selections compared with the stronger limit by the stable feeding.

### Temperature variations in the printed parts

When printing samples 1 to 4 at increasing traveling speeds *v*, the temperature in the center on edge BC on the second layer (Fig. 2b) was measured, and the corresponding temporal profiles are presented in Fig. 6a on a shifted time line . The time origin takes the deposition time at each chosen location. A magnified part of the profile upon deposition is shown in Fig. 6b, where it is clear that the initial deposition temperature (extrudate temperature at the nozzle outlet $\bar{T}$) decreases with increasing *v*. The maximum drop in the deposition temperature is 10.1℃ (compared with that at the lowest *v*), which is only slightly higher than the counterpart result by the CFD simulations (7.7℃, Table 2). These observations partially validate the CFD model in the study.

After the deposition, the temperature profiles at different *v* overlap with each other to a great extent before their respective interlayer reheating. The temperature upon reheating (indicated by black dots in Fig. 6a) is higher at a higher *v*, because the cooling duration is shorter when the interlayer reheating time Δτ is reduced. Interlayer reheatings take place first at t=8.89 s for v=13.5 mm/s (sample 4), then at t=12 s for v=10 mm/s (sample 3), …, and lastly at t=24 s for v=5 mm/s (sample 1). These characteristics in the temporal temperature profiles are correctly predicted by simulations in Fig. 6c.

However, certain discrepancies still exist between the monitoring and simulations: the IR readings are slightly higher than the numerical results, which are ascribed to both the monitoring errors and the simulation uncertainties with respect to geometries, process parameters, and material properties. To explain and minimize such discrepancies is off the main theme in this study, but readers can refer to the Advanced Manufacturing Lab^32^ for more information.

Apart from the profile overlapping, both the monitoring and simulations strongly imply that, for instance, at the nozzle traveling speed v=10 mm/s, should the interlayer reheating disappear, the temperature profile in Fig. 6a, would follow the path as the case of v=5 mm/s. Such implications can also arise from temperature profiles measured with thermocouples.^35^ These observations postulate that the cooling rate in FFF is independent of the traveling speed, disregarding the weak dependence of the deposition temperature on the traveling speed in the stable feed region.

Fundamentally, neither the thermal conduction, convection, nor radiation depends on *v* (ignoring the radiant heat gain from the hot-end^36^). Nevertheless, to print a given part, *v* determines Δτ. Once the interlayer reheating happens, the thermal boundary changes, and the temperature profile will be different. Ultimately, *v* is observed to be correlated to the part cooling globally; but on a local time frame before the interlayer reheating, the part cooling is intrinsically independent of *v*.

To verify the postulation that the instantaneous cooling rate is independent of the traveling speed, sample 5 (L=60 mm, v=10 mm/s) was printed, in contrast to sample 1 (L=30 mm, v=5 mm/s). Apart from *v*, all other independent process parameters remained identical (filament feed speed is not an independent variable). In printing the two samples, the interlayer time was the same $\Delta \tau =\frac{4L}{v}=$24 s. The experimental and numerical temporal temperature profiles on the second layer are presented in Fig. 6d. The simulations predicted them to be identical. The monitoring confirmed such resemblance. Overall, the experimental profile of sample 5 was only slightly lower than that of sample 1. This difference can be partially explained by the negative dependence of the deposition temperature on the flow rate (Fig. 6b, or Table 2), while the remaining deviations are within measurement errors of the IR camera.

Based on these observations, an *interlayer time similarity* rule is proposed

$$\begin{array}{cc} & T_{1}\left(x,z,t;v_{1},L_{1}\right)=T_{2}\left(cx,z,t;v_{2},L_{2}\right),when\frac{L_{2}}{L_{1}}=\frac{v_{2}}{v_{1}}=c.\\ & \left[interlayertimesimilarity\right] \end{array}$$

This rule states that temperature fields in printing two single-walled squares can be identical when the interlayer time is the same. The similarity originates from the independence of local cooling rate on the traveling speed, and can extend to other single-walled geometries. This rule suggests that the cooling in FFF is primarily determined by the thermal boundaries of the deposited strands. Thus, parameters (such as the traveling speed) having no/weak influence over the thermal boundaries should have no/weak influence over the transient cooling before the interlayer reheating. The authors also confirmed this interlayer time similarity rule with different setups in different processes (layer height range from 0.6 to 1.4 mm, volume flow rate range from 14.2 to 302 cm^3^/h) with different materials (PLA, homemade PLA/Flax fiber filament, homemade PLA/glass fiber filament).^32^

In practice, this interlayer time similarity rule shows that temperature information obtained in printing small geometries can be transferred to print big objects, and the traveling speed is not a primary limitation in large-scale printing. Hence, it provides an efficient tool to guide trial-and-error explorations, especially when FFF is controlled by the robot arm to print big objects at high volume flow rates.

### Critical insights

#### An example of scale-up production

According to the interlayer time similarity rule, the nozzle traveling speed could have be raised to v=27 mm/s when printing sample 5 (L=60 mm). Also, a successful print is expected, based on the temperature of sample 4 (L=30 mm, v=13.5 mm/s, Fig. 6a). The effects of deceleration and acceleration at sharp corners are disregarded. However, such printing is not recommended: on the v−¯h plane in Fig. 4b, this process parameter combination is in the unstable region (marked by ×). Correspondingly, a designed volume flow rate $Q_{v}^{\prime }=$76.6 cm^3^/h would be required, but it is apparently beyond the maximum stable flow rate possible with the current E3D print head assembly. Bounded by the inequality (14), the maximum nozzle traveling speed is 15.0 mm/s. However, when the nozzle temperature is set to 210℃, the maximum speed could be raised to 18.6 mm/s. Compared with the current settings in printing sample 5, the production efficiency is higher by 27%. To further increase the efficiency, using a more powerful print head assembly will be fundamental. The key findings in this article (the similarity rule, extrudate temperature reduction, the maximum stable volume flow rate) can be translated in printing at higher volume flow rates or layer heights. The authors also conducted a series of experiments confirming these results, in the layer height range from 0.6 to 1.4 mm and volume flow rate range from 14.2 to 302 cm^3^/h, on different hot-end setups (E3D and Dyze Design Typhoon^®^), and with different materials.

#### Predict the temperature before printing

Sample 4 of edge length L=30 mm was successfully printed at speed v=13.5 mm/s. Nevertheless, signs of slow-cooling-induced surface irregularities were observed (Fig. 2b). To minimize such printing defects while maintaining the production efficiency, the layer height ¯h and traveling speed *v* can be varied along the black-dashed line in Fig. 4b (activating the part cooling fan is also a solution). Aiming for a lower temperature upon interlayer reheating, should a smaller layer height be used? It is widely accepted that a smaller ¯h benefits faster cooling^37^; but to remain at the same efficiency, *v* has to be raised to ensure a constant volume flow rate, leading to a reduction in the interlayer time, which is correlated to a higher temperature before the interlayer reheating as observed in Fig. 6a. To solve the puzzle, the numerical model from the Advanced Manufacturing Lab^32^ can reveal how the cooling/temperature before reheating will be affected, as demonstrated in Supplementary Appendix Figure SA2. In this study, choosing a smaller layer height can lead to a lower temperature before the interlayer reheating (while simultaneously offering a lower surface roughness). However, this is not a universal answer in every process, because the temperature variations can be more sensitive to the changes in interlayer time as well (Supplementary Appendix Fig. SA2).

## Conclusions

This article provides a systematic and complete analysis of the temperature development in FFF, from the filament entering the hot-end to the printed parts. A standard E3D print head assembly from a desktop FFF printer was mounted on a robot arm for printing. The setup realized the immediate need for part geometry scalability, but it is not necessarily ideal for scale-up production due to the limit in material throughput. Accordingly, a stable working region of the hot-end was identified with a limit in the volume flow rate for reliable material feeding. This limit sets an upper bound for the nozzle traveling speed at a given layer height. Working at the highest stable volume flow rate guarantees the highest production efficiency, but not necessarily a successful printing, this later being affected by the temporal and spatial temperature variations in the printed parts. With simulations and thermal monitoring, it was also found that at a higher nozzle traveling speed, the temperature became less homogeneous inside the hot-end, and the extrudate temperature deviated further from the nominal nozzle temperature. However, the cooling in the deposited part is intrinsically independent of the traveling speed, as long as comparable thermal boundaries are guaranteed in different processes. Ultimately, an interlayer time similarity rule was formulated for printing single-walled structures, which states that the temperature fields are comparable for processes sharing the same interlayer time. This rule suggests that knowledge obtained in printing small objects can be transferred to print big objects, and thus, it provides direct guidance over scale-up productions.

## Supplementary Material

Supplemental data

Supplemental data

## Data Availability

The numerical model for temperature simulations in the printed parts is available from the Advanced Manufacturing Lab.^32^ Other data that support the findings of this study are available from the corresponding authors upon reasonable request.

## Supplementary Material

Supplementary Appendix SA1

Supplementary Appendix SA2

## List of Selected Symbols

*A* strand cross-section area [mm^2^]
*c_p_* specific heat capacity at constant pressure [$\frac{J}{g\deg C}$]
*d* filament diameter [mm]
*d*_1_ barrel diameter [mm]
*h* convective heat transfer coefficient [Wm2 degC]
¯h layer height/layer thickness [mm]
*k* thermal conductivity [$\frac{W}{m\deg C}$]
*L* sample edge length [mm]
*p* pressure [Pa]
Pe dimensionless Péclet number
$Q_{v}$ measured volume flow rate/throughput [cm^3^/h]
$Q_{v}^{\prime }$ designed volume flow rate/throughput [cm^3^/h]
*t* time [s]
T temperature $T=T\left(x,z,t\right)$ [℃, K]
$T_{n}$ nominal nozzle temperature [℃]
$T_{\infty }$ room (far environment) temperature [℃, K]
$\bar{T}$ extrudate temperature at nozzle outlet/deposition temperature [℃]
*u*_0_ inlet speed at the barrel entrance in the CFD simulation [mm/s]
***u*** velocity vector [mm/s]
*v*_0_ filament feed speed [mm/s]
*v* nozzle traveling speed [mm/s]
α thermal diffusivity [mm^2^/s]
Δτ interlayer time [s]
ε material emissivity
$\varepsilon_{\lambda }$ wavelength-specific emissivity in thermography
η shear viscosity [Pa·s]
ξ shape factor of the strand cross section
ρ mass density [g/cm^3^]
ϕ nozzle diameter [mm]
