# Supplementary material for: Temperature Analyses in Fused Filament Fabrication: From Filament Entering the Hot-End to the Printed Parts
Source: 3D Print Addit Manuf. 2022 Apr 11;9(2):132–42. doi: 10.1089/3dp.2020.0339 (PMC9831539; doi:10.1089/3dp.2020.0339)
Supplement: Supplemental data [file Supp_DataSA1.docx]

**A1** Shear viscosity of the material

Prior to the viscosity measurement, the filament was cut into granulates of $\sim$2 mm in length and dried at 80 ℃ for four hours. The Cox-Merz relation was assumed valid for the shear rate range in FFF (the maximum apparent wall shear rate $\frac{32Q_{v}^{'}}{\pi\phi^{3}}$ in the nozzle was on the order of 100 s^-1^), and thus used to convert complex viscosity to the shear viscosity. At different test temperatures (190, 200, 210, 220 ℃), the shear viscosity was fitted to the Carreau-Yasuda model

$$\eta\left( \dot{\gamma} \right)=\eta_{\infty}{+(\eta_{0}-\eta_{\infty})\left[ 1+\left( \lambda\dot{\gamma} \right)^{a} \right]}^{\frac{n-1}{a}},$$

where, $\eta_{0}$ and $\eta_{\infty}$ are the viscosity at the zero and infinite shear rate, respectively, $\lambda$ is the relaxation time, $\dot{\gamma}$ the shear rate, $n$ the power law index, and $a$ the transition index. **FIG.A1** shows that the model provides a good fit in both the high viscosity and the transition region. The fitting results are given in Table **A1**. In the CFD model, an Arrhenius type temperature dependence was implemented for the viscosity with a factor $f\left( T \right)=e^{\frac{B}{T+273.15}-\frac{B}{T_{r}+273.15}}$, where the parameter $B=$ 9500 ℃ was taken at the reference temperature $T_{r}=$ 200 ℃.

**FIG.A1** Shear viscosity of the PLA filament at different temperatures and shear rates.

**Table A1** The Carreau-Yasuda model fitting results for the shear viscosity of PLA.

| Temperature  [℃] | Viscosity at Zero Shear $\eta_{0}$ [mPa·s] | Viscosity at Infinite Shear $\eta_{\infty}$ [mPa·s] | Relaxation Time $\Delta\lambda$ [s] | Transition Index $a$ | Power Law index $n$ |
| --- | --- | --- | --- | --- | --- |
| 190 | 3.726$\times$10^6^ | 0.1 | 1.906$\times$10^-2^ | 0.6913 | 0.2812 |
| 200 | 2.399$\times$10^6^ | 0.1 | 2.139$\times$10^-2^ | 0.7955 | 0.3587 |
| 210 | 1.537$\times$10^6^ | 0.1 | 1.506$\times$10^-2^ | 0.8273 | 0.4002 |
| 220 | 1.049$\times$10^6^ | 0.1 | 1.407$\times$10^-2^ | 0.9285 | 0.4685 |
