# Supplementary material for: Temperature Analyses in Fused Filament Fabrication: From Filament Entering the Hot-End to the Printed Parts
Source: 3D Print Addit Manuf. 2022 Apr 11;9(2):132–42. doi: 10.1089/3dp.2020.0339 (PMC9831539; doi:10.1089/3dp.2020.0339)
Supplement: Supplemental data [file Supp_DataSA2.docx]

**A2** The temperature before inter-layer reheatings in different processes, when the volume flow rate is fixed at 38.3 cm^3^/h.

**FIG.A2** The temperature before the inter-layer reheatings at different layer height $\hbar$. At each $\hbar$, the travelling speed $v$ is scaled so that a constant volume flow rate is maintained ($\hbar v=$ 38.3 cm^3^/h). The temperature was taken from the centre of edge BC on the 5^th^ layer in the simulations. For current sample #4 ($\hbar=$ 0.6 mm, $v=$ 13.5 mm/s), the temperature before reheating is more sensitive to layer height, thus choosing a smaller $\hbar$ can deliver a faster cooling.
